# COVID-19-related severe MS exacerbation with life-threatening Takotsubo cardiomyopathy in a previously stable patient and interference of MS therapy with long-term immunity against SARS-CoV-2

**DOI:** 10.1007/s00415-021-10779-0

**Published:** 2021-10-07

**Authors:** Brigitte Wildemann, Sven Jarius, Lorenz H. Lehmann, Florian André, Norbert Frey, Paul Schnitzler, Laura Jäger, Christoph Gumbinger, Andrea Viehöver

**Affiliations:** 1 Molecular Neuroimmunology Group, Department of Neurology, University of Heidelberg, Im Neuenheimer Feld 400, 69120 Heidelberg, Germany; 2 Department of Cardiology, Angiology and Pneumology, University of Heidelberg, Im Neuenheimer Feld 410, 69120 Heidelberg, Germany; 3 Department of Infectious Diseases/Virology, University of Heidelberg, Im Neuenheimer Feld 324, 69120 Heidelberg, Germany; 4 Department of Neurology, University of Heidelberg, Im Neuenheimer Feld 400, 69120 Heidelberg, Germany

Dear Sirs,

Little is known about how infection with SARS-CoV-2 impacts the course and severity of multiple sclerosis (MS). Here, we report on a patient in whom SARS-CoV-2 infection was followed by severe exacerbation of previously stable MS and Takotsubo cardiomyopathy (TTC). Of note, repeated intravenous pulse therapy with high-dose methylprednisolone (IVMP), plasma exchange, and administration of ocrelizumab interfered with the development of a long-lasting immune response against SARS-CoV-2.

*Case report.* In a 39-year-old woman, relapsing remitting MS was first diagnosed in 2011 and had been stable since 2014 with continuous administration of dimethyl fumarate (DMF). In December 2020, 10 days after the onset of marked general malaise and fatigue, the patient developed nystagmus, dizziness and headache. The next day she experienced tachycardia, followed the day after by coughing and shortness of breath requiring admission to hospital. Chest CT showed multifocal central and peripheral ground-glass opacities involving both pulmonary lobes suggesting COVID-19 pneumonia. PCR testing of a throat swab was positive for SARS-CoV-2-RNA and negative for influenza virus. Laboratory tests and ECG screening revealed elevated hs-troponinT (715 pg/ml; normal < 14) and intermittent ventricular bigeminy, respectively. Cardiac catheterization showed no coronary artery disease but regional wall motion abnormalities compatible with atypical TTC. Cranial MRI depicted a symptomatic novel lesion in the dorsomedial medulla oblongata (Fig. 1a, d). The patient was diagnosed with COVID-19 pneumonia, TTC and acute MS relapse. She was treated with oxygen, beta-blocking and angiotensin converting enzyme-inhibiting agents, and IVMP 1000 mg daily for 4 days. Four weeks after symptom onset, the patient noticed sudden deterioration of her general condition along with irregular heart rhythm as well as marked dizziness, double vision, and difficulties on swallowing. Cardiac MRI findings and a renewed increase in hs-troponinT serum levels (163 pg/ml) were again compatible with TTC (supplemental material). On neurologic assessment, she presented with dysarthria, severe dysphagia requiring tube feeding and marked gait instability. Repeat cranial MRI showed several new brain lesions and enlargement of the brainstem lesion, now contrast-enhancing (Fig. 1b, c, e–g). Cell-based testing for serum myelin oligodendrocyte glycoprotein (MOG)-IgG [3, 6, 7] and aquaporin-4 (AQP4)-IgG [5] was negative. Cerebrospinal fluid analysis disclosed 4 cells/µl, mild blood–CSF barrier dysfunction, and CSF-restricted oligoclonal IgG bands. The antibody indices for measles and varicella zoster viruses were 2.07 and 1.67, respectively, indicating a positive MRZ reaction, as typically seen in MS but not in MOG encephalomyelitis or AQP4-IgG-positive neuromyelitis optica[1, 2, 4]. CSF-PCR for HSV1/2, varicella zoster virus, and SARS-CoV-2 was negative, as was serology for CSF and serum SARS-CoV-2-IgG. IVMP therapy (5 × 2000 mg daily) and a course of seven plasma exchanges prompted slow improvement of the clinical symptoms. DMF was stopped and replaced by ocrelizumab to control MS disease activity. Lymphocyte counts were normal (> 1/nl) during therapy with DMF (03/2019: 1.5/nl, 04/2020: 1.7/nl, 10/2020: 2/nl) and at the time of SARS-CoV-2 infection (12/2020: 2.5/nl). Repeat serology for SARS-CoV-2-IgG performed after the last plasma exchange and again 38 days later (i.e., 22 days after ocrelizumab) was negative.

**Fig. 1:**
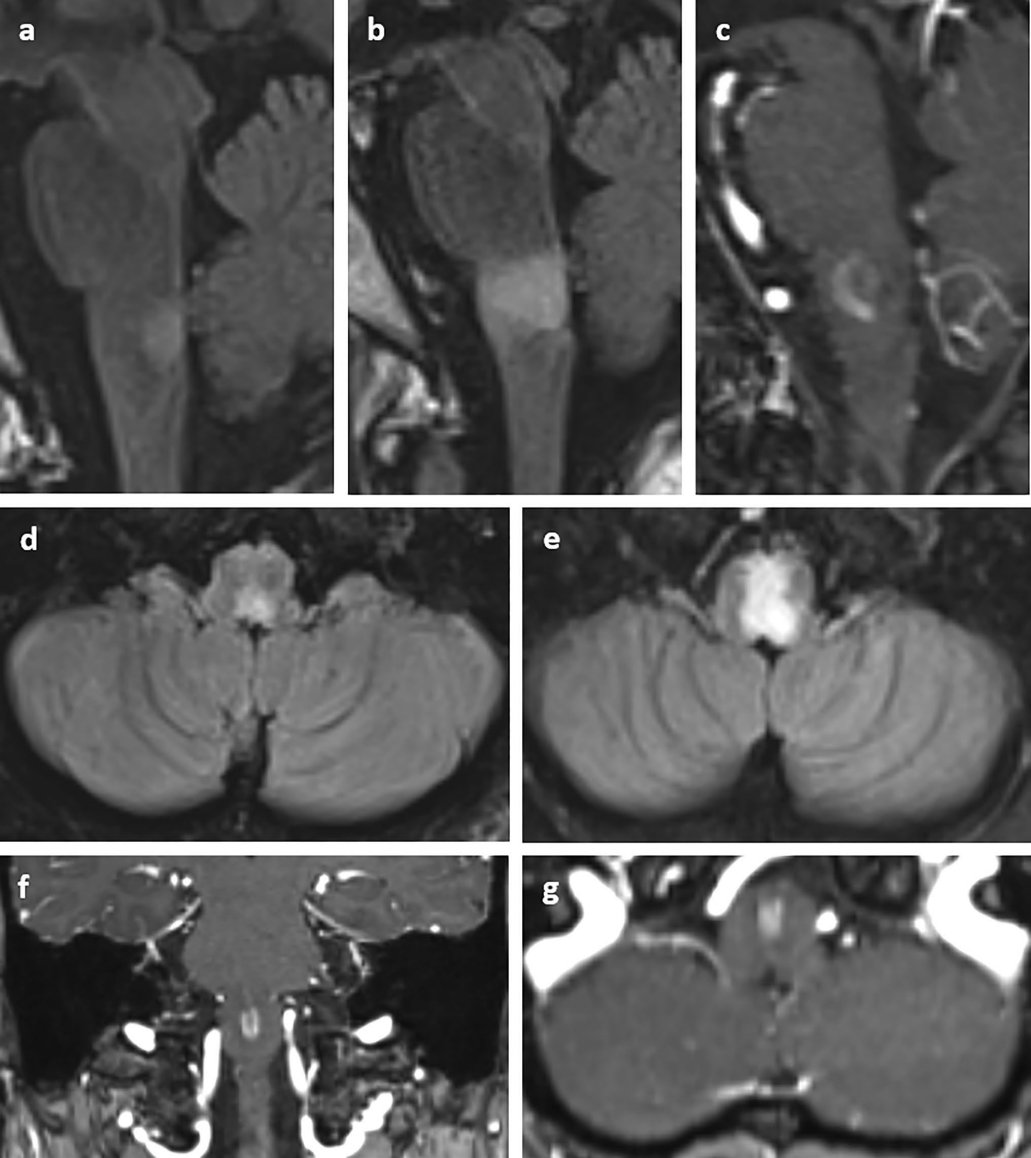
Cranial MRI 5 days after the onset of neurological symptoms revealed a T2/FLAIR-hyperintense lesion without gadolinium enhancement (not shown) in the dorsomedial medulla, localized in the nucleus tractus solitarii (**a**) Follow-up MRI 25 days later at the time of neurological deterioration showed enlargement of the same lesion (**b**), now with gadolinium enhancement (**c**). **d**, **e** Corresponding T2/FLAIR-weighted axial sections at day 5 after symptom onset and 25 days later. Panels **f**, **g** Gadolinium enhancement of the lesion at follow-up MRI in coronal (**f**) and axial (**g**) sections

*Discussion.* This patient developed severe MS activity 10 days after the onset of COVID-19 symptoms. The risk of MS relapses is elevated between 2 weeks before and 5 weeks after the first symptoms of an infection, and viral respiratory tract infections are thought to be the most important triggers[10]. Thus, it appears likely that recent infection with SARS-CoV-2 prompted the pronounced clinical and radiological disease activity in this patient, whose MS had been well controlled for many years on treatment with dimethyl fumarate. This illustrates that SARS-CoV-2 infection might considerably harm MS patients by provoking true MS exacerbation in addition to infection-related pseudo-relapses[12]. Of note, the patient had no cardiac comorbidities and denied emotional stress prior to experiencing the cardiac arrhythmia that preceded neurological deterioration. Thus, although TTC—a disorder prompted by a surge in catecholamine levels—has been described as a cardiac complication of COVID-19 [13], altogether it appears more likely that the acute demyelinating lesion in the dorsomedial medulla arising due to breakthrough MS disease activity was responsible for the myocardial dysfunction. The dorsomedial medulla includes the nucleus of the solitary tract, an inhibitory vasomotor center that regulates and dampens sympathetic outflow. Injury to this structure in the context of MS brainstem exacerbations has been reported to cause acute cardiopulmonary events including TTC [8, 9, 11, 14]. In line with previous reports, the course of COVID-19 was moderate overall despite long-term immunotherapy and was also not negatively affected by additional IVMP treatment. In conclusion, this case (i) demonstrates that COVID-19 might be capable of triggering severe and even life-threatening MS exacerbations and suggests that patients with MS should be particularly well protected from SARS-CoV-2 infection; (ii) supports previous evidence suggesting that MS-related brainstem lesions can cause TTC; (iii) arouses concerns regarding the establishment of long-term humoral immunity against COVID-19 in patients treated for MS with plasma exchange, ocrelizumab, steroids and/or other immunotherapies during acute SARS-CoV-2 infection (although a sufficient cellular immune response may still develop, as suggested by the overall favorable outcome from COVID-19 in our patient).

## Supplementary Information

Below is the link to the electronic supplementary material.

Supplemental video 1. Left ventricular two-chamber view showing akinesia of the midventricular myocardial segments. Cine images were acquired using a steady-state free precession sequence with 35 phases per cardiac cycle (WMV 908 kb)
